# A UAV Vision-Based Deformation Monitoring Method with 3D Scale Constraints

**DOI:** 10.3390/s25247418

**Published:** 2025-12-05

**Authors:** Jianlin Liu, Jun Wu, Wujiao Dai, Deyong Pan, Min Zhou, Lei Xing, Zhiwu Yu

**Affiliations:** 1 National Engineering Research Center of High-Speed Railway Construction Technology, Changsha 410075, China; 2 China Railway Group Limited, Beijing 100039, China; 3 School of Geosciences and Info-Physics, Central South University, Changsha 410083, China

**Keywords:** 3D scale, UAV, vision measurement, deformation monitoring

## Abstract

**Highlights:**

**What is the main finding?**

- The study developed a spatial 3D scale that provides high-precision scale infor-mation and proposed a UAV visual deformation monitoring method with 3D scale constraints.

**What are the implications of the main findings?**

- This method effectively addresses the scale difference between the survey area model and the real model caused by low-quality images and low-precision control points, meeting the needs of high-precision engineering defor-mation monitoring.
- The spatial 3D scale has low production cost and simple on-site deployment, which enhances the applicability of UAV visual deformation monitoring and pro-vides a reliable solution for large-scale deformation monitoring.

**Abstract:**

Aiming at the problem that low-quality images and low-precision control points lead to scale differences between the survey area model and the real model in UAV (Unmanned Aerial Vehicle) vision-based 3D deformation monitoring, which impairs the accuracy of deformation monitoring, this paper develops a spatial 3D scale for providing high-precision scale information and proposes a UAV vision-based deformation monitoring method with 3D scale constraints, thereby improving the deformation monitoring accuracy in large-scale survey areas. Experimental results show that compared with the monitoring method using only control points as constraints, the proposed method achieves accuracy (RMSE) improvement rates of 38.6% and 48.1% in the horizontal and elevation directions respectively during four phases of UAV operations, and the 3D deformation accuracy (RMSE) improvement rate remains at approximately 42.3% during seven phases of UAV operations. This verifies the effectiveness and reliability of the UAV vision-based deformation monitoring method with 3D scale constraints.

## 1. Introduction

Vision measurement is a method that uses computer vision technology to analyze and process images or videos to obtain information such as the position, shape, and size of target objects. It features non-contact, low cost, and high precision, and is often used for deformation monitoring of structures such as bridges and buildings [1,2]. With the rapid development of UAV hardware and software systems, the performance of UAVs has been greatly enhanced, and research on UAV combined with vision measurement has continued to deepen in fields such as landslide disaster identification [3] and deep foundation pit deformation monitoring [4]. Structure from Motion (SfM) [5,6] is a method that detects and tracks feature points from multi-view image sequences and reconstructs 3D models based on the visual motion relationships between feature points. This method can quickly obtain 3D models of measured objects and has been widely applied in model construction and 3D detection of slopes, coastal cliffs, tunnels, and other scenarios [7,8,9,10]. Currently, UAV-based 3D deformation monitoring combined with SfM is mostly for census-type disaster detection or low-precision centimeter-level or decimeter-level deformation monitoring [11,12]. However, according to the “GB50026-2020 Engineering Surveying” standard [13], the accuracy requirements for deformation-sensitive high-rise buildings, high slopes, etc., are 0.5 mm for elevation mean error and 3.0 mm for point position mean error, thus failing to meet the needs of millimeter-level high-precision engineering deformation monitoring applications. Existing studies have shown that low image quality [14,15,16] and restricted control point layout [17,18] are important factors affecting the accuracy of measurement area model reconstruction. In UAV-based visual 3D deformation monitoring, this is manifested as follows: (1) During UAV operations, complex conditions such as illumination changes, occlusion, and short exposure time cause low-quality UAV image data (e.g., overexposure, underexposure, blurriness, shadow occlusion), which affects the extraction of feature information in the measurement area and the detection and positioning of various markers, thereby impacting the accuracy of the measurement area model and final deformation monitoring; (2) Due to potential deformation hazards inside the deformation monitoring area, control points can only be arranged in stable areas at the edge of the measurement area, resulting in sparse and uneven distribution of control points, insufficient control effect on the measurement area model, and reduced deformation monitoring accuracy. The impact of the above problems on the accuracy of 3D visual deformation monitoring is largely reflected in the scale difference between the generated model and the real model. To make the constructed model closer to the model with real physical lengths, scholars have conducted relevant research in the field of industrial photogrammetry, using high-precision scale rulers to determine the length benchmark of the measurement coordinate system, thereby improving the measurement accuracy of the system [19]. Among them, 3D calibration targets mostly adopt regular geometric structures (such as cubic frames), with known distances between feature points guaranteed by high-precision processing, suitable for close-range and small-scale precision measurement scenarios. However, their complex structure and large mass make it difficult to flexibly arrange them in large-scale outdoor measurement areas, and under the high-altitude shooting perspective of UAVs, the feature points of the targets are prone to occlusion or reduced recognition accuracy. Multi-scale measuring rods provide scale benchmarks through single or multiple fixed-length rod structures, featuring simple structure and strong portability, but can only provide scale constraints in a single direction, failing to achieve synchronous calibration of horizontal and elevation directions, and it is difficult to solve the scale ambiguity problem. Moreover, in the oblique photography scenario of UAV SfM, the measuring rods have poor stability when placed obliquely, and the feature points at both ends lack clear identification markers, leading to dilution of scale accuracy. Therefore, it is necessary to develop a more applicable scale ruler model for the application background of UAV vision deformation monitoring to provide higher-precision scale conversion information and achieve higher-precision model reconstruction and deformation monitoring.

To address the above problems, this paper first designs and develops a spatial 3D scale ruler for providing high-precision scale conversion coefficients, and proposes a UAV vision deformation monitoring method with 3D scale constraints to achieve higher-precision deformation monitoring in large-scale areas. Compared with 3D calibration targets in industrial photogrammetry, the spatial 3D scale ruler designed in this paper adopts a lightweight orthogonal structure of “two lower and one upper”, balancing structural stability and flexibility in outdoor layout; compared with multi-scale measuring rods, it provides multiple sets of scale constraints in horizontal and elevation directions simultaneously through three orthogonal rod systems, effectively improving the comprehensiveness and reliability of scale calibration. Finally, experiments are conducted to verify the effectiveness and feasibility of the UAV vision deformation monitoring method with 3D scale constraints.

## 2. Materials and Methods

### 2.1. Development of Three-Dimensional Spatial Ruler

In the field of UAV measurement, control points can provide high-precision position information for model reconstruction and establish the connection between images and real-world coordinates. However, under measurement conditions where the layout of control points is difficult, the accuracy of model reconstruction cannot be guaranteed, failing to meet the requirements of high-precision UAV 3D deformation monitoring. To improve the accuracy of the survey area model, a ruler with a known length can be placed in the survey area to calculate the scale conversion coefficient, thereby making the scale of the survey area model closer to that of the real model. Existing rulers are mostly rod-shaped structures, which cannot be reliably and stably placed in a tilted manner in the survey area, resulting in insufficient constraints in the vertical direction. Moreover, in close-range photogrammetry scenarios with a flight altitude of several tens of meters, the two ends of the ruler lack effective identification features, and the precision of the ruler itself is greatly diluted.

**Figure 1:**
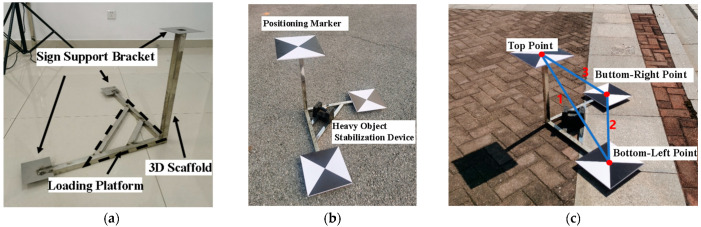
Spatial 3D Scale Ruler. (**a**) Spatial 3D Bracket; (**b**) Spatial 3D Scale Ruler Model; (**c**) Ruler Edges.

Therefore, based on project requirements, this study designs a spatial 3D scale ruler suitable for UAV vision-based deformation monitoring scenarios with flight heights of 25 m and 50 m, considering aspects such as structural relationships, dimensions, and identification effects. The specific design is as follows: To enable the ruler to provide constraint information in multiple directions, three rods with a length of 0.5 m are used to form the main body of the ruler in a pairwise orthogonal manner, and the ruler is designed in a “two lower, one upper” structure—i.e., two rods form a supporting surface, and one rod is placed vertically to provide height information. Meanwhile, a load-bearing platform is built between the two rods of the supporting surface for placing weights to stabilize the ruler; visual positioning markers for identification are fixed at the endpoints of the three rods. In this study, cross-shaped markers with clear centers are adopted, which have good visual effects during image processing and facilitate the accurate positioning of ruler points. Based on the above design, the spatial 3D scale ruler model shown in Figure 1 is obtained. Through the three ruler edges arranged in space in the ruler model, higher-precision scale information in the horizontal and vertical directions is provided, thereby improving the accuracy of model reconstruction and deformation monitoring.

In UAV vision monitoring, the Ground Sampling Distance (GSD) directly determines the identifiability of scale ruler markers and the positioning accuracy of endpoints, representing the ground size corresponding to each pixel. The GSD calculation formula is as follows:

(1) $$\mathrm{GSD}=\frac{Hu}{f}$$

where H is the UAV flight height (m), u is the pixel size (μm), and f is the focal length of the onboard vision sensor (mm). To adapt to higher flight heights, the marker size or rod length can be adjusted to maintain a fixed proportional relationship with GSD. For example, when the flight height changes, the scale ruler rod length and marker size need to be adjusted proportionally to the flight height to ensure the stability of scale constraint accuracy.

As shown in Figure 1a, the spatial 3D bracket consists of a 3D bracket main body, marker support brackets for fixing positioning markers, and a load-bearing platform for stabilizing the ruler model. The spatial 3D scale ruler model built with the spatial 3D bracket can realize simultaneous constraints in the horizontal and vertical directions, and one spatial 3D scale ruler model can provide 3 sets of known length constraints, thereby achieving higher scale precision and effectively avoiding accidental errors caused by a single ruler. In the spatial 3D scale ruler, the upper point forms the 1st and 3rd ruler edges with the lower left point and lower right point respectively, and the lower left point and lower right point form the 2nd ruler edge, as shown in Figure 1c. Assuming that N spatial 3D scale rulers are arranged in one measurement, there are 3N ruler edges in total. For the nth spatial 3D scale ruler (0 < n ≤ N), the following relationships exist:

(2) $$\left\{\begin{array}{l} S_{n1}=L_{n1}/l_{n1}\\ S_{n2}=L_{n2}/l_{n2}\\ S_{n3}=L_{n3}/l_{n3} \end{array}\right.$$

where $L_{\mathrm{ni}}(i\in \left[1,\;2,\;3\right])$ represents the actual measured length of the ith ruler edge in the nth ruler, $l_{\mathrm{ni}}$ represents the length of the ith ruler edge in the nth ruler under the model coordinate system, and $S_{\mathrm{ni}}$ represents the conversion coefficient from the model coordinate system to the real-world coordinate system calculated based on the ith ruler edge in the nth ruler. Furthermore, the average scale conversion coefficient $S_{n}$ of the nth ruler is calculated according to Formula (2).

(3) $$\left\{\begin{array}{l} S_{n}=\alpha_{n1}S_{n1}+\alpha_{n2}S_{n2}+\alpha_{n3}S_{n3}\\ 1=\alpha_{n1}+\alpha_{n2}+\alpha_{n3} \end{array}\right.$$

where $\alpha_{\mathrm{ni}}$ represents the weight coefficient of $S_{\mathrm{ni}}$ in the calculation of $S_{n}$ and $\alpha_{n1}$, $\alpha_{n2}$, $\alpha_{n3}$ are all set to 1/3. After obtaining the average scale conversion coefficient of each scale, the final scale conversion coefficient S is calculated according to Formula (3).

(4) $$\left\{\begin{array}{l} S=\beta_{1}S_{1}+\beta_{2}S_{2}+\ldots +\beta_{n}S_{n}\\ 1=\beta_{1}+\beta_{2}+\ldots +\beta_{n} \end{array}\right.$$

where $S_{j}(j\in \left[1,\;2\ldots \;N\right])$ represents the scale conversion coefficient of the jth ruler, $\beta_{j}$ represents the weight coefficient of $S_{j}$ in the calculation of S and its value is 1/N. After obtaining the scale conversion coefficient S, the world coordinates $P_{\mathrm{ws}}(X_{\mathrm{ws}},Y_{\mathrm{ws}},Z_{\mathrm{ws}})$ of point p after scale conversion are calculated according to Formula (4).

(5) $$\left[\begin{array}{c} X_{ws}\\ Y_{ws}\\ Z_{ws} \end{array}\right]=S\left[\begin{array}{c} X_{w}\\ Y_{w}\\ Z_{w} \end{array}\right]$$

where $\left(X_{w},Y_{w},Z_{w}\right)$ is the world coordinate of point p without scale correction. Through Formula (4), more accurate coordinates of monitoring points can be obtained, thereby improving the accuracy of deformation monitoring.

This paper adopts isotropic scale conversion, i.e., it is assumed that the scale distortion of the model coordinate system and the real-world coordinate system is consistent in the X, Y, and Z dimensions, and global scaling is achieved through a single factor S. The rationality of this assumption is based on the following premises: (1) The three sets of constraint edges of the spatial 3D scale ruler cover the horizontal and elevation directions, which can comprehensively reflect the overall scale deviation of the 3D space. (2) In SfM model reconstruction, the scale uncertainty caused by calibration errors of camera internal parameters is mostly manifested as globally consistent distortion rather than significant anisotropic differences. Although the complete 7-parameter similarity transformation can theoretically solve the anisotropic problem, it will increase the complexity of the model and the difficulty of parameter solution. In this paper, through the layout of multiple sets of 3D scale rulers and the average design of multiple constraint edges, local anisotropic errors have been offset to a certain extent. Moreover, the scale accuracy under the isotropic assumption can meet the requirements of deformation detection accuracy.

#### Error Propagation Model

The error sources affecting the monitoring coordinate results include scale length error $∆L_{\mathrm{ni}}$ and endpoint positioning error $∆l_{\mathrm{ni}}$. According to Equations (1)–(3) and the law of error propagation, we can obtain:

(6) $$\sigma_{S}^{2}={\left(\frac{1}{N}\right)}^{2}\sum_{j=1}^{N}\left[{\left(\frac{1}{3}\right)}^{2}\sum_{i=1}^{3}\left(\frac{\sigma_{L_{ji}}^{2}}{l_{ji}^{2}}+\frac{L_{ji}^{2}\sigma_{1_{ji}}^{2}}{l_{ji}^{4}}\right)\right]$$

simplified as:

(7) $$\sigma_{S}\approx \frac{1}{3N}\sqrt{\sum_{j=1}^{N}\sum_{i=1}^{3}\left(\frac{\sigma_{L}^{2}}{l_{ji}^{2}}+\frac{L_{ji}^{2}\sigma_{l}^{2}}{l_{ji}^{4}}\right)}$$

From Equation (4), the coordinate error variance is obtained:

(8) $$\sigma_{P_{wS}}^{2}=P_{w}^{2}\sigma_{S}^{2}+S^{2}\sigma_{P_{w}}^{2}$$

where $\sigma_{s}$ is the scale coefficient error, and $\sigma_{P_{w}}$ is the coordinate error without scale correction. Therefore, the scale length error and endpoint error are transmitted to the final coordinates through the scale coefficient, and the coordinate error is jointly affected by the scale length error and endpoint error.

### 2.2. Control Point Constraints

**Figure 2:**
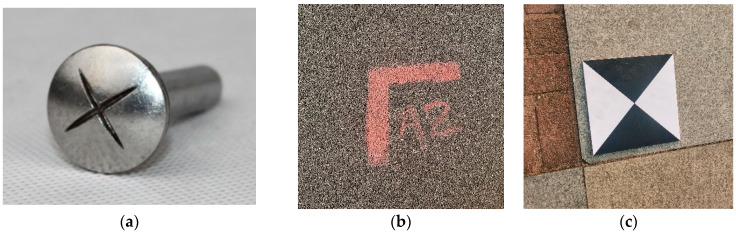
Deployment Forms of Control Points. (**a**) Survey nail; (**b**) Spray-painted marker; (**c**) Marker board.

Ground control points are important factors in UAV 3D model construction, with known 3D coordinates and accuracy information, which can be used to correct the distortion and positioning errors of UAV images and provide the conversion relationship between image coordinates and real coordinates. They are usually arranged in the measurement area in the form of survey nails, spray painting, signboards, etc. (as shown in Figure 2).

Assuming that the measurement area model has k control points and t images, the camera positioning optimization objective function shown in Equation (9) is constructed:

(9) $$g\left(X,R\right)={\sum_{i=1}^{k}\sum_{j=1}^{t}\rho_{\mathrm{ij}}\left\|P\left(x_{i},R_{j}\right)-q_{ij}\right\|}^{2}$$

where $\rho_{\mathrm{ij}}$ represents the positional relationship between control point i and image j. If control point i has a projection point in image j, $\rho_{\mathrm{ij}}$ is 1; otherwise, it is 0. $x_{i}$ represents the world coordinates of control point i, and $R_{j}$ represents the camera parameters corresponding to the shooting of image j. The function P projects control point i onto image j, and minimizes the distance from the real projection point $q_{\mathrm{ij}}$ while optimizing $x_{i}$ and $R_{j}$, so that the error between the projection position of the control point in the image and its real position is minimized, and the optimized camera position and attitude are obtained.

By arranging a certain number of reasonably distributed control points in the measurement area, the accuracy of model reconstruction can be improved, and the accuracy of monitoring results can be guaranteed. Generally, when selecting control points, the following principles should be followed:

(1) Clear position. Cross-shaped markers with clear center positions are used as the position carriers of control points. Compared with markers such as building corners and intersections selected in non-visual ways, they have more identifiable features, and their structure is conducive to computer algorithm recognition, with greater advantages in automated position extraction.
(2) Uniform distribution. Control points should be evenly distributed in the measurement area to avoid concentrated layout in a certain area, ensuring the accuracy of subsequent 3D modeling.
(3) High point accuracy. The coordinate measurement results of control points should have high precision and reliability, and total stations, GNSS measurement equipment, and RTK receiving equipment can be used for control point position measurement.

**Figure 3:**
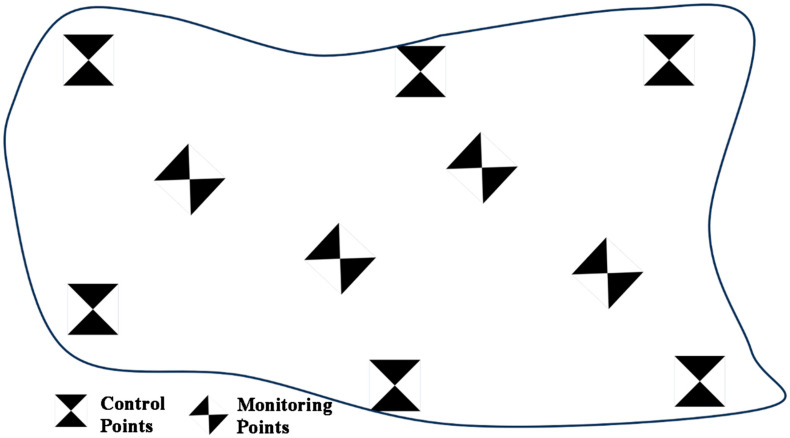
Schematic Diagram of Control Point Deployment.

As shown in Figure 3, due to potential deformation hazards inside the measurement area, the requirements for control point layout cannot be met. In this paper, control points are evenly arranged in stable areas at the edge of the measurement area, and different control points are distinguished manually to provide the best constraint effect. Marker monitoring points are arranged in the hazard area inside the measurement area for accurate and rapid positioning of monitoring points, realizing the monitoring of hazard points in the deformation area.

### 2.3. Workflow of the UAV Vision-Based Deformation Monitoring Method with 3D Scale Constraints

The general workflow of UAV vision-based deformation monitoring using the spatial 3D scale ruler is as follows:

(1) Layout of points in the survey area: Arrange the spatial 3D scale rulers in unobstructed areas, plan the UAV operation route, and conduct UAV image acquisition.
(2) Image quality evaluation: Disable low-quality images (image quality lower than 0.7) in subsequent processing and reconstruct a rough model using UAV POS data.
(3) Use Metashape software (v. 2.2.1) to manually locate the positions of feature points such as control points and scale ruler points, construct spatial 3D scale rulers, calculate the scale conversion coefficient according to Equation (3), and scale the generated model to the real-world coordinate system.
(4) Use the adaptive Radon transform marker detection and positioning method to perform high-precision repeated positioning of the markers in step (3) to obtain high-precision and highly consistent marker coordinates.
(5) Export the 3D coordinates of monitoring points in the current phase. Repeat steps (1) to (4) to obtain the 3D coordinates of monitoring points in the next phase, and calculate the difference between the coordinates of the two phases to obtain the deformation value of the two phases of monitoring.

**Figure 4:**
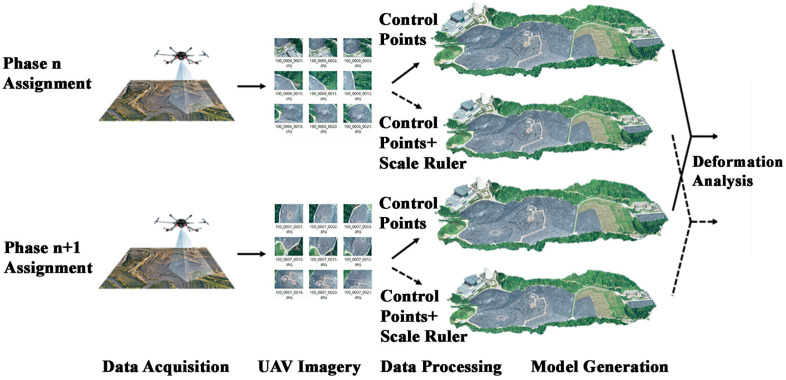
Workflow of UAV Vision-based Deformation Monitoring with 3D Scale Constraints.

Based on the above workflow, the brief steps of the conventional processing method and the 3D scale constraint-based processing method are shown in Figure 4.

This paper uses an adaptive Radon transform-based detection and positioning method [20] for UAV displacement measurement markers to locate cross-shaped markers. The root mean square error (RMSE) of marker detection and positioning by this method under different combinations of flight heights, marker information collection radii, and marker sizes is 0.57 pixels. The specific positioning process is as follows:

(1) Image collection: UAV data collection.
(2) Calculation of detection parameters: After obtaining information (marker size W, UAV flight height H, ratio s of the center line length to the side length of the marker, focal length f and pixel size u of the UAV vision sensor), calculate the marker information collection radius R and the edge width L of the cross-shaped scoring template, where the calculation formulas of R and L are:
  (10) $$\left\{\begin{array}{l} R=\frac{Wsf}{2Hu}\\ L=\frac{\left(D-\sqrt{D^{2}-k}\right)}{2} \end{array}\right.$$
  where $D=\sqrt{2}(2R+1)$.
(3) Marker detection and positioning: Obtain a saliency map using the Radon transform, locate peak points through surface fitting, and finally obtain the marker positioning results.

### 2.4. Calculation of Deformation Monitoring Results

After reconstructing the survey area model, assume n monitoring points are deployed in the survey area. For the mth monitoring point, its 3D coordinates in the ith and jth monitoring phases are $\left(X_{\mathrm{mi}},Y_{\mathrm{mi}},Z_{\mathrm{mi}}\right)$ and $\left(X_{\mathrm{mj}},Y_{\mathrm{mj}},Z_{\mathrm{mj}}\right)$, respectively. The displacement $D_{\mathrm{ij}}^{m}$ of the mth monitoring point between the ith and jth phases is calculated via Equation (11):

(11) $$D_{\mathrm{ij}}^{m}=\sqrt{{\left(X_{mj}-X_{mi}\right)}^{2}+{\left(Y_{mj}-Y_{mi}\right)}^{2}+{\left(Z_{mj}-Z_{mi}\right)}^{2}}$$

If the true displacement $d_{\mathrm{ij}}^{m}$ of the mth monitoring point between the ith and jth phases is known, the monitoring error $e_{\mathrm{ij}}^{m}$ of the jth monitoring point is calculated using Equation (12):

(12) $$e_{\mathrm{ij}}^{m}=D_{\mathrm{ij}}^{m}-d_{\mathrm{ij}}^{m}$$

After calculating the monitoring error of each monitoring point, the overall deformation monitoring accuracy $R_{\mathrm{ij}}$ between the ith and jth phases is obtained via Equation (13):

(13) $$Rij=\sqrt{\frac{{\sum_{m=1}^{n}\left(e_{\mathrm{ij}}^{m}\right)}^{2}}{n}}$$

## 3. Experiment Design and Result

### 3.1. UAV 3D Deformation Monitoring Experiment

**Figure 5:**
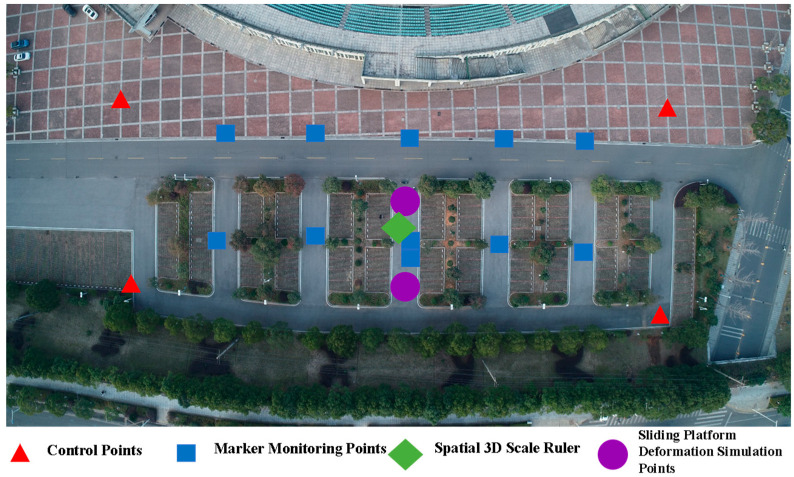
Schematic Diagram of Experimental Point Layout.

This section mainly verifies the performance and feasibility of the UAV vision-based deformation monitoring method with 3D scale constraints. The proposed method is compared with two other deformation monitoring methods—one using only control points and the other using control points plus a single ruler as constraints—to analyze the accuracy and effectiveness of the proposed method. Scholar [21] explores the impact of ground control point layout on deformation monitoring accuracy, and the experimental results show that arranging control points evenly around the measurement area and having control points in the internal area is more in line with the overall control idea. However, due to the nature of the deformation monitoring experiment, to prevent internal deformation from affecting the results, it is best not to arrange control points in the internal measurement area. As shown in Figure 5, the experimental site is an area of approximately 10,081.8 square meters located directly south of the stadium in the new campus of Central South University. To simulate a real monitoring scenario, 4 marked control points are arranged at the edge of the survey area, 11 marked monitoring points inside the survey area, 2 3D sliding table deformation simulation points, and 1 spatial 3D scale ruler.

The spatial 3D scale ruler is placed in the central area of the measurement area to achieve uniform propagation of 3D scale constraints. In addition, to improve the accuracy in the elevation direction, the scale ruler model needs to be placed obliquely to better provide elevation constraints. Only 4 control points are arranged at the edge of the measurement area, and the middle area lacks direct constraints, while the marker monitoring points and deformation simulation points for deformation monitoring are all distributed inside the measurement area. Placing the 3D scale ruler in the middle of the measurement area, the 3 sets of known length constraints it provides can radiate evenly to the surroundings, enabling the model scale of each area in the measurement area to be close to the true value and avoiding scale deviation in the middle area caused by edge constraints. If the scale ruler is moved to the edge of the measurement area, the central monitoring points far from the scale ruler cannot effectively obtain scale constraints, and the difference between the model and the real scale will increase with distance, especially the constraint effect in the elevation direction will decay sharply.

**Figure 6:**
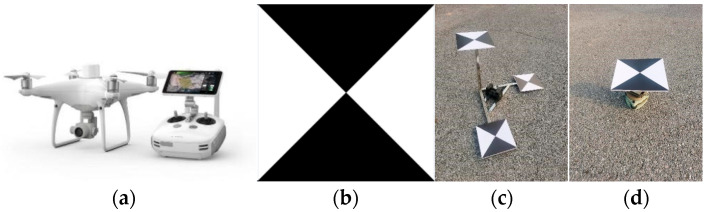
Experimental Equipment and Materials. (**a**) Phantom 4 RTK; (**b**) Markers; (**c**) Spatial 3D Scale Ruler; (**d**) 3D Sliding Table.

The various equipment used in the experiment is shown in Figure 6. Among them, the UAV used is the DJI Phantom 4 RTK (DJI-Innovations, Shenzhen, China), a small multi-rotor high-precision aerial survey UAV equipped with a centimeter-level navigation and positioning system, a high-performance imaging system, and professional route planning applications. It can carry out monitoring work quickly and flexibly at a relatively low cost; the measurement markers used in the experiment are cross-shaped with a size of 25 cm × 25 cm, which have the advantages of easy layout and easy identification; the spatial 3D scale bar is fixed in an undisturbed area within the survey region via a weight stabilization device. Constructed from 304 stainless steel, it exhibits excellent corrosion resistance and heat resistance, with a pipe length tolerance of ±0.5 mm. The side lengths of the scale bar are measured using a total station, achieving a design accuracy of ±1 mm; the main body of the 3D sliding table deformation simulation points is a three-axis sliding table device with scales, and a platform for placing markers is designed on the top of the sliding table. By moving the sliding table by a known distance in different monitoring periods as the preset deformation value, this value is used as an indicator for checking accuracy during result analysis, with an accuracy of 1 mm. The control points are measured using a Leica TS09 total station, with an accuracy of 2.2 mm.

The lengths of each ruler edge of the spatial 3D scale ruler used in the experiment are shown in Table 1:

A total of 3 groups of deformation monitoring operations were conducted in this experiment (4 consecutive phases of UAV operations, and each two consecutive phases of UAV operations constitute one group of deformation monitoring operations). For each phase of UAV operation, 2 groups of dynamic displacement checks and 12 groups of static displacement checks were designed. The dynamic displacement checks were performed using 2 3D sliding table deformation simulation points; the static displacement checks included 11 monitoring marker points arranged in the survey area and 1 distance check value composed of two markers (located in the center of the survey area shown in Figure 3, with a known distance between the centers of the two markers and an accuracy of 1 mm). The theoretical deformation displacement of the monitoring marker points is 0. In the experimental result analysis, 14 groups of check indicators were jointly used for accuracy evaluation.

In this experiment, the sparse point cloud reconstruction step was implemented using the low-quality alignment function of Metashape software to simulate the scenario of poor observation conditions in real UAV operations and explore the accuracy improvement performance of the proposed method under low image quality conditions. The information of UAV operations in each phase is shown in Table 2:

#### Experimental Results and Analysis

The collected image data were processed using the control point method and the control point + 3D scale constraint method respectively, and the processing results of the deformation simulation points in this experiment are shown in Table 3.

As shown in Table 3, in some cases, the absolute error of the proposed method is larger than that of the control point method. For example, the horizontal monitoring error of deformation simulation point 1 in the third group (3.5 mm) is slightly higher than that of the control point method (3.4 mm). This is because the imaging conditions during UAV operations are complex and changeable, and the quality of some collected images is poor, thus affecting the accuracy of some experimental results. In addition, in the 2nd and 3rd groups of experiments on Deformation Simulation Point 2, the absolute error of the proposed method in the elevation direction is relatively large, exceeding 20 mm. This is also due to environmental changes during UAV flight that result in variations in image quality and poor data quality, leading to insufficient precision in elevation direction feature extraction and thus significant errors. However, overall, compared with the control point method, the control point plus 3D scale constraint method has smaller measurement errors in horizontal and elevation directions, verifying the effectiveness of the spatial 3D scale ruler in improving monitoring accuracy.

The statistical results of mean absolute error, standard deviation, and RMSE in horizontal and elevation directions are as follows.

The calculation formulas of various errors are as follows, with RMSE adopted as the core accuracy indicator in this study.

(14) $$\sigma =\sqrt{\frac{1}{n-1}\sum_{i=1}^{n}{\left(E_{i}-\bar{E}\right)}^{2}}$$

(15) $$RMSE=\sqrt{\frac{1}{n}\sum_{i=1}^{n}E_{i}^{2}}$$

It can be seen from Table 4 that the mean absolute error, standard deviation, and RMSE of the proposed method in horizontal and elevation directions are significantly lower than those of the traditional control point method, with higher and more stable monitoring accuracy. Due to the lack of internal constraints in the control point method, the error distribution is extremely uneven, with abnormal errors. In contrast, the proposed method performs multi-directional constraints through the spatial 3D scale ruler, which can comprehensively correct scale errors.

**Figure 7:**
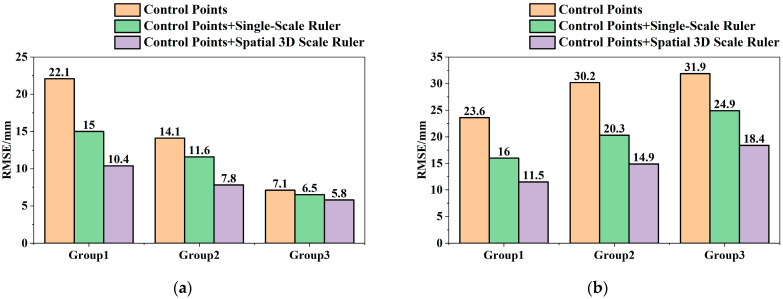
Experimental Results. (**a**) Horizontal Monitoring Accuracy of Different Rulers; (**b**) Vertical Monitoring Accuracy of Different Rulers.

To explore the accuracy improvement performance of the spatial 3D scale ruler compared with a single scale ruler, the data were processed using the control point plus single scale ruler constraint method, where the single scale edge uses the No. 2 scale edge of the spatial 3D scale ruler, and the processing results are shown in Figure 7.

Based on the experimental results shown in Figure 7, the accuracy improvement rate was calculated according to Formula (16):

(16) $$\left\{\begin{array}{c} AIR_{H}=\frac{A_{H}-A_{H}^{\prime }}{A_{H}}\times 100\%\\ AIR_{E}=\frac{A_{E}-A_{E}^{\prime }}{A_{E}}\times 100\% \end{array}\right.,$$

where AIR represents the accuracy improvement rate; the subscripts H and E represent the horizontal and vertical directions respectively; A represents the deformation monitoring accuracy (RMSE) of the control point constraint method; $A^{\prime }$ represents the deformation monitoring accuracy (RMSE) after adding constraint conditions. The calculation results are shown in Table 4.

It can be seen from Table 5 that under the condition of low image quality, both the deformation monitoring methods of control points + single ruler constraint and control points + 3D scale constraint can improve the monitoring accuracy. Due to the certain slope of the survey area ground and the tilted image acquisition of the UAV camera, the method of ground control points + single ruler constraint can improve the monitoring accuracy in both horizontal and vertical directions, but the accuracy is still at a relatively low level; the control points + 3D scale constraint method has a significantly better accuracy improvement rate in both horizontal and vertical directions than the single ruler constraint method, achieving the best accuracy improvement effect among the three methods in both directions, with the vertical accuracy improvement effect reaching 48.1%, which verifies the constraint effect of the spatial 3D scale ruler model in the vertical direction.

It can be seen from Table 6 that the RMSE of the 3D scale ruler is higher than that of the single-scale ruler method in both horizontal and vertical directions. The reason is that the multi-constraints of the 3D scale ruler impose higher requirements on data quality, leading to the amplification of coupling errors. Consequently, the dispersion of the accuracy improvement rate and the overall error increase significantly. In contrast, the single-scale ruler relies on a single reference, which is less affected by coupling interference, while its upper limit of accuracy improvement is relatively lower.

In conclusion, the experimental results of this study show that the proposed UAV vision-based deformation monitoring method with control points and 3D scale constraints can effectively improve the deformation monitoring accuracy in both horizontal and vertical directions; compared with the control point method and the control point + single ruler constraint method, the proposed method achieves better accuracy improvement effects in both horizontal and vertical directions, which verifies the practicality and effectiveness of the proposed method in improving monitoring accuracy.

### 3.2. Landslide Monitoring Experiment at the Northwest Corner of Chuangyuan Primary School in Kaifu District, Changsha City

**Figure 8:**
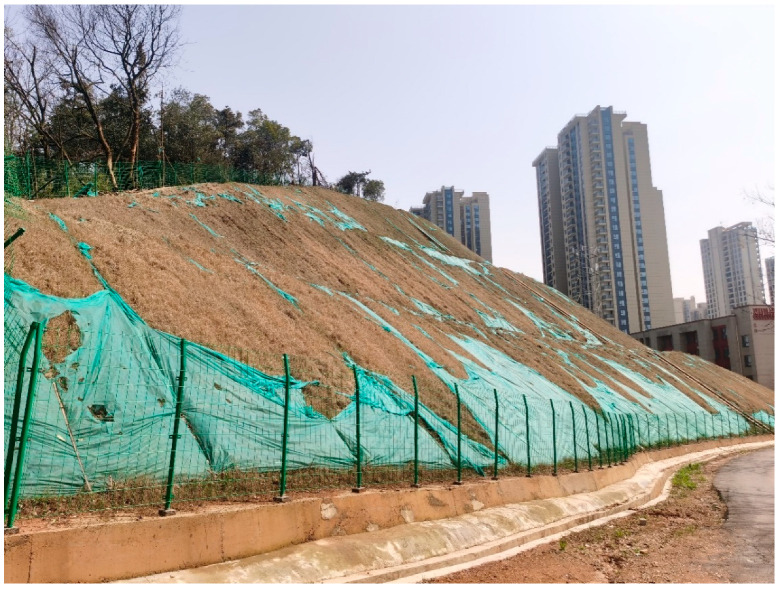
Actual Map of the Landslide at the Northwest Corner of Chuangyuan Primary School.

To further verify the performance of the multi-constraint method, a deformation monitoring experiment was conducted on the landslide at the northwest corner of Chuangyuan Primary School in Xinglian Community, Xiufeng Subdistrict, Kaifu District, Changsha City. The landslide is located in a hilly area with undulating terrain, an altitude of 60~80 m, and a relative height difference of about 20 m (as shown in Figure 8). A sudden geological disaster occurred here on the afternoon of 2020-05-18, damaging the construction access road at the bottom of the slope and threatening the safety of teachers and students of Chuangyuan Primary School below the slope and pedestrians passing on the construction access road below. Therefore, it is of great significance to carry out planned monitoring and early warning for this landslide to ensure the safety of school teachers and students and road pedestrians.

**Figure 9:**
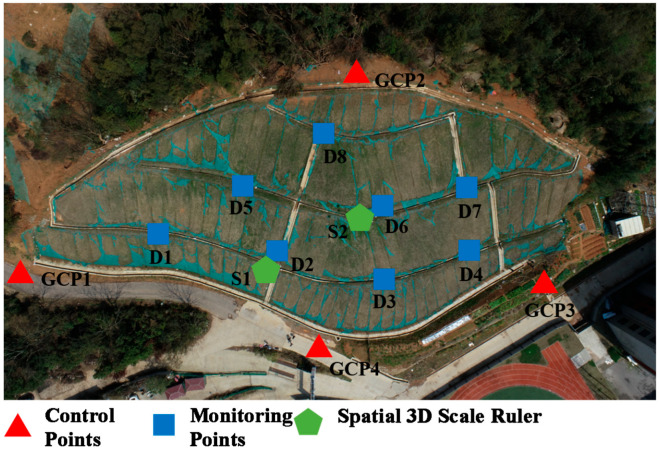
Schematic Diagram of Point Layout for the Landslide at the Northwest Corner of Chuangyuan Primary School.

Considering that the landslide is a small-scale landslide, this scheme plans to arrange 4 marker control points, 8 marker monitoring points, and 2 spatial 3D scale rulers, and the detailed layout diagram of the points is shown in Figure 9.

**Figure 10:**
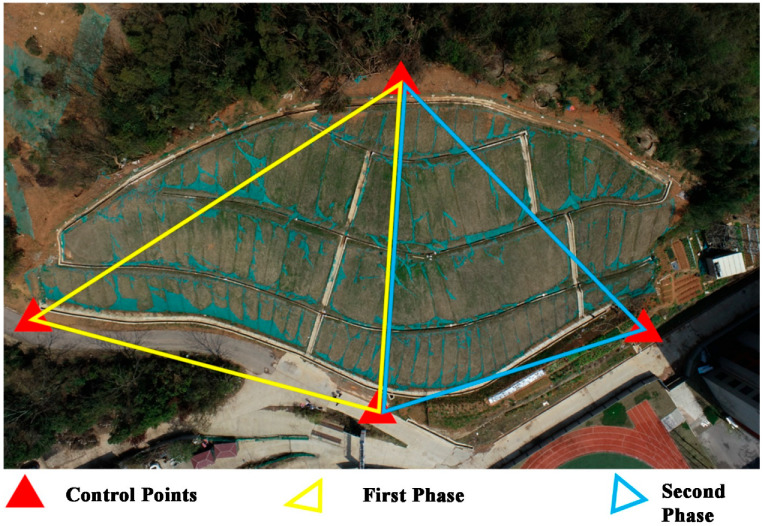
Static Measurement Network Diagram of Control Points.

In this experiment, the DJI Phantom 4 RTK UAV was used for data collection, and four specifications of markers (40 cm × 40 cm, 35 cm × 35 cm, 30 cm × 30 cm, and 25 cm × 25 cm) were used for point layout. Among them, 3 control points (GCP1, GCP3, and GCP4) near the slope bottom used 40 cm × 40 cm markers, monitoring points D1~D4 and spatial 3D scale ruler S1 used 35 cm × 35 cm markers, monitoring points D5~D7 and spatial 3D scale ruler S2 used 30 cm × 30 cm markers, and monitoring point D8 and control point GCP2 used 25 cm × 25 cm markers. The markers were fixed on concrete with strong foam adhesive. As shown in Figure 10, static measurement was used for control point measurement, with 2 measurement periods and 120 min per period, and the control point measurement accuracy was 1.4 mm.

**Figure 11:**
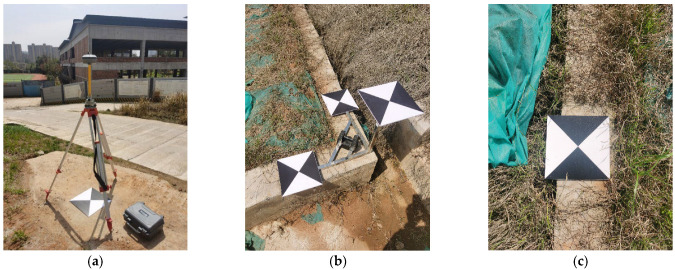
Field Diagram of Experimental Instruments and Equipment. (**a**) GNSS Receiver; (**b**) Spatial 3D Scale Ruler; (**c**) Maker.

Some instruments and equipment used in the experiment are shown in Figure 11:

A total of 6 groups of deformation monitoring operations were conducted in the experiment (7 consecutive UAV operations, and each two consecutive UAV operations form 1 group of deformation monitoring operations). Since all experimental data were collected in the afternoon of the same day, it is considered that all monitoring points had no displacement, and all monitoring points were used as check points with a displacement value of 0 for accuracy evaluation. Taking the slope bottom as the benchmark, the UAV flight height was 50 m, the flight speed was set to 2.0 m/s, the longitudinal/transverse overlap rate was 85%/80%, and the camera tilt angle was −60 degrees. The experimental weather was sunny (20 °C) with northwest wind level 2. In the experimental processing, sparse point cloud reconstruction was realized by low-quality alignment of Metashape software to simulate the situation of poor UAV observation conditions and explore the monitoring performance of the multi-constraint method under low image quality conditions.

#### Experimental Results and Analysis

**Figure 12:**
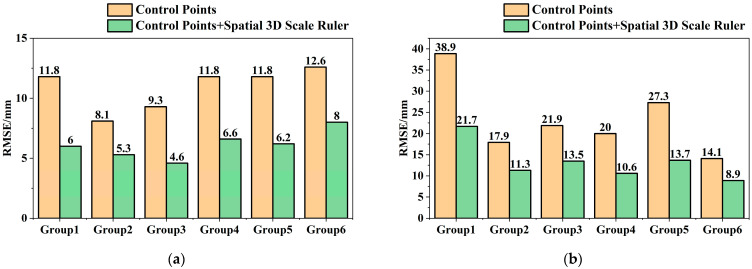
Comparison of Accuracy between Control Point Method and Multi-Constraint Method. (**a**) Comparison of Horizontal Accuracy between Control Point Method and Multi-Constraint method; (**b**) Comparison of Elevation Accuracy between Control Point Method and Multi-Constraint Method.

The 3D coordinates of monitoring points in each phase were calculated using the control point method and the multi-constraint method respectively, and the deformation monitoring results between each two phases were calculated according to the 3D coordinates of monitoring points. Secondary analysis was conducted on the deformation monitoring result data to obtain the accuracy (RMSE) of the control point method and the multi-constraint method in horizontal and elevation directions, as shown in Figure 12.

**Figure 13:**
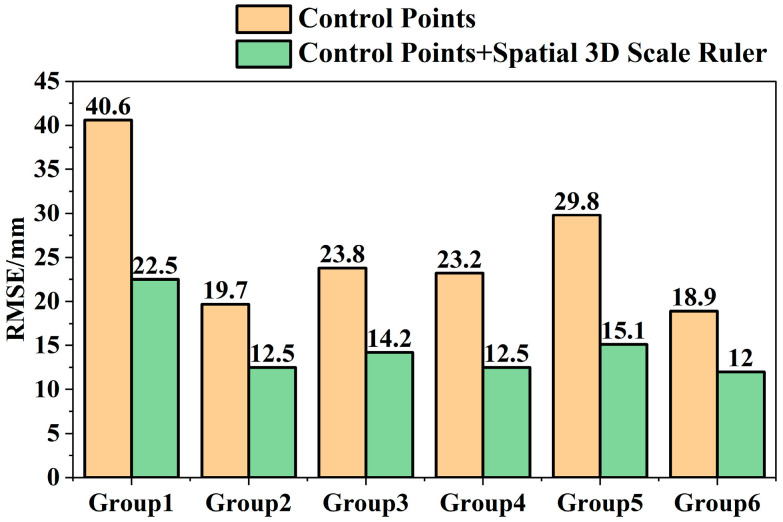
Comparison of 3D Displacement Deformation Monitoring Accuracy between Control Point Method and Multi-Constraint method.

To intuitively express the difference in displacement monitoring accuracy between the two methods, the comprehensive 3D displacement deformation of the two methods was calculated according to Equation (5), as shown in Figure 13.

The 3D deformation accuracy (RMSE) improvement rate was calculated according to Equation (5), and the calculation results are shown in Table 7.

It can be seen from Table 7 that under the condition of low-quality UAV image data, the accuracy of the multi-constraint deformation monitoring method is better than that of the control point method, and the accuracy improvement rate remains at approximately 42.3%, verifying the effectiveness of the multi-constraint deformation monitoring method. The overall standard deviation of the six groups of data is 0.053, indicating good stability in the accuracy improvement of this method.

## 4. Discussion

To address the scale discrepancy between the measurement area model and the real model in UAV vision-based deformation monitoring, this study designs and develops a spatial 3D scale capable of providing high-precision scale information, thereby proposing a 3D scale-constrained UAV vision deformation monitoring method. Experimental results demonstrate that the proposed method, by combining control points with 3D scale constraints, can effectively improve deformation monitoring accuracy, and its precision enhancement effect is more significant compared to traditional methods. Meanwhile, the spatial 3D scale features low manufacturing cost, convenient on-site deployment, and strong applicability, which can meet the practical needs of some high-precision engineering deformation monitoring scenarios.

Currently, the academic community mainly controls scale errors by optimizing ground control point (GCP) layout and improving camera network design, but both approaches have obvious limitations. For GCP layout optimization, when there are deformation hazards in the measurement area, GCPs cannot be deployed in the central region, leading to a significant decline in precision improvement. Additionally, adding more GCPs increases measurement costs and operational intensity, making it difficult to adapt to large-scale and complex terrain monitoring scenarios. For camera network design improvement, in low-quality image scenarios, merely adjusting parameters such as overlap rate and flight height is insufficient to completely resolve the scale distortion issue in the elevation direction. In contrast, the 3D scale-constrained method proposed in this study, based on reasonable GCP deployment and optimized camera network design, possesses three core advantages: (1) Low operational cost: High-precision monitoring can be achieved only by deploying scales inside the measurement area. (2) Synchronous constraint in horizontal and elevation directions through a 3D orthogonal structure, effectively solving the scale ambiguity problem in the elevation direction during UAV monitoring. (3) Independence from GCP layout constraints: It maintains monitoring precision relying on its own scale reference, making it suitable for scenarios where GCP deployment is difficult.

However, the phenomenon that the absolute elevation error exceeds 20 mm in the 2nd and 3rd groups of Deformation Simulation Point 2 also exposes the shortcomings of the method: Firstly, although the spatial 3D scale is designed with a precision of 1 mm, the thermal expansion and contraction characteristics of stainless steel pipes (especially during outdoor temperature fluctuations) are prone to causing deviations in the actual scale length. Moreover, the positioning error of the cross-shaped markers is transmitted to the monitoring point coordinates through the scale conversion process, ultimately leading to error accumulation. Secondly, the assumption of isotropic scale conversion has theoretical limitations. In real scenarios, the scale distortion of the model coordinate system in the X, Y, and Z dimensions is not completely consistent, and the elevation direction is more significantly affected by factors such as terrain undulation, making it difficult for a single scaling factor to fully offset anisotropic errors. Thirdly, the types of experimental sites are relatively single, covering only flat campus areas and hilly landslide sites with limited environmental complexity. In contrast, actual engineering deformation monitoring scenarios involve various terrains such as deep foundation pits and high-steep slopes, and the performance of the proposed scale and method in these complex scenarios has not been verified, resulting in certain application limitations. Fourthly, the UAV flight height in the experiments is fixed at 25 m and 50 m, and the spatial 3D scale has a single size. For ultra-large-scale measurement areas, multiple scales need to be deployed, making it difficult to flexibly adapt to different measurement area sizes and deformation monitoring precision requirements.

To address the above shortcomings, future research will carry out optimizations from multiple aspects: Firstly, expand experimental scenarios by conducting verification in complex terrains such as deep foundation pits and high-steep slopes to test the adaptability of the method in different environments. Secondly, develop multi-size spatial 3D scales that can be flexibly adjusted according to the measurement area size and precision requirements, reducing the deployment cost for ultra-large-scale measurement areas. Thirdly, optimize scale performance and marker positioning algorithms: select more stable materials for scale fabrication to reduce the impact of environmental factors, and adopt higher-precision marker positioning technologies to fundamentally reduce the influence of scale length errors and endpoint positioning errors on coordinate results. Fourthly, improve scale design for different application scenarios: for example, add counterweights on steep or rugged slopes to enhance stability; use corrosion-resistant and low-temperature-resistant materials with infrared markers in high-altitude areas; strengthen 3D constraints through multi-scale deployment in ultra-large-scale measurement areas; and adjust the scale rod length and marker size proportionally with flight height to ensure the stability of scale constraint precision. Fifthly, in scenarios with uneven GCP distribution, reasonably deploy 3D scales in GCP-dense and GCP-sparse areas, and balance the scale precision of different regions through scale conversion coefficients. In the future, the UAV 3D vision deformation monitoring method will be further improved through the above optimizations to expand its engineering application prospects.

## Figures and Tables

**Table 1:** Lengths of Ruler Edges.

Name of Ruler Edge	Length (m)
1	0.7185
2	0.6980
3	0.7194

**Table 2:** UAV operation information.

Flight Altitude	Flight Speed	GSD	Overlap Rate (Longitudinal/Lateral)	Camera Model	Camera Resolution	Camera Focal Length	Camera Tilt Angle
25 (m)	2.4 (m/s)	0.68 (cm/pixel)	80%/75%	FC6310R	5472 × 3648	8.8 (mm)	−60°

**Table 3:** Preset Displacement Values and Measured Values.

Monitoring Group	Deformation Simulation Point 1			Deformation Simulation Point 2		
	Group 1	Group 2	Group 3	Group 1	Group 2	Group 3
Preset Horizontal Displacement (mm)	7.1	14.1	10.0	5.0	10.0	15.0
Horizontal Displacement by Control Point Method (mm)	15.6	12.1	6.6	37.8	29.0	12.7
Horizontal Displacement by Proposed Method (mm)	8.8	13.8	6.5	21.8	21.9	13.0
Horizontal Absolute Error by Control Point Method (mm)	8.5	2.0	3.4	32.8	19	2.3
Horizontal Absolute Error by Proposed Method (mm)	1.7	0.3	3.5	16.8	11.9	2.0
Preset Vertical Displacement (mm)	−9.0	9.0	0.0	0.0	0.0	0.0
Vertical Displacement by Control Point Method (mm)	8.8	−12.3	30.8	42.0	−51.6	32.1
Vertical Displacement by Proposed Method (mm)	−22.4	20.5	7.8	18.0	−30.7	22.6
Vertical Absolute Error by Control Point Method (mm)	17.8	21.3	30.8	42.0	51.6	32.1
Vertical Absolute Error by Proposed Method (mm)	13.4	11.5	7.8	18.0	30.7	22.6

**Table 4:** Error Statistics of Different Methods.

Monitoring Direction	Method	$\mathrm{Mean}\;\mathrm{Absolute}\;\mathrm{Error}\;\bar{E}$ (mm)	Standard Deviation σ (mm)	RMSE (mm)
Horizontal	Control Point Method	11.33	12.3	15.90
	Proposed Method	6.03	6.70	8.59
Vertical	Control Point Method	32.6	12.65	34.58
	Proposed Method	17.33	8.30	18.92

**Table 5:** Accuracy Improvement Performance of Various Methods (Compared with the Control Point Method).

	Control Points + Single Ruler		Control Points + Spatial 3D Scale Ruler	
	Horizontal	Vertical	Horizontal	Vertical
Group 1	32.1%	32.2%	52.9%	51.3%
Group 2	17.7%	32.8%	44.7%	50.7%
Group 3	8.5%	21.9%	18.3%	42.3%
Average	19.4%	29.0%	38.6%	48.1%

**Table 6:** Error Statistics of Accuracy Improvement Rates.

Monitoring Direction	Method	Standard Deviation σ (%)	RMSE (%)
Horizontal	Control points + single scale Ruler	11.8	21.7
	Control points + spatial 3D scale Ruler	17.6	41.4
Vertical	Control points + single scale Ruler	6.13	29.4
	Control points + spatial 3D scale Ruler	5.03	48.3

**Table 7:** Three-dimensional Deformation Accuracy Improvement Rate of Multi-Constraint Method (Compared with Control Point Method).

Monitoring Group	Accuracy Improvement Rate (%)
Group 1	44.7
Group 2	36.6
Group 3	40.3
Group 4	45.9
Group 5	49.3
Group 6	36.8
Average	42.3

## Data Availability

The original contributions presented in this study are included in the article. Further inquiries can be directed to the corresponding author.

## Abbreviations

The following abbreviations are used in this manuscript:

UAV	Unmanned Aerial Vehicle
SfM	Structure from Motion
3D	Three-Dimensional
GSD	Ground Sampling Distance
